# Medical Opinion and Movement

**Published:** 1907-06-15

**Authors:** 


### Medical Opinion and Movement.

The addition of spirit to water has been credited with the power to destroy any impurity that may exist in the water. This, however, has been shown to be quite fallacious. The bactericidal power of alcohol of ordinary drinking strength is insignificant or *nil*. This appears not to be the case, however, for wines. MM. Sabrazès and Marcandier have shown that wines, white wines more than red, are quite powerful to destroy the typhoid bacillus. But this power is due, not to the contained alcohol, but to the acids of the wines, and apparently varies in proportion to their acidity.

We are so accustomed to associate our American brethren with startling and novel ideas that we can hardly be surprised if even the medical man, in spite of the cold philosophy of his scientific training, succumbs to the prevalent charm of the sensational. It was perhaps on this account that some credence was given to the report in the lay Press that Dr. Knopf, of New York, speaking at the National Tuberculosis Congress at Washington, advised his hearers "to kill their dying consumptives quickly and painlessly by heavy doses of morphine," and was liberally applauded. We are glad, therefore, to be able to state that this is only one of the many perversions of the truth to which our lay contemporaries are so liable when they attempt to deal with technical, and especially medical or scientific, questions. In a letter addressed to the medical journals by Dr. George Dock, chairman of the meeting, he most emphatically denies that any such remark was made by Dr. Knopf. Dr. Knopf simply advocated the use of morphine to relieve painful symptoms in the last stage of the disease, and in that sense only was he understood by members of the Congress present.

We are now regaled with another sensational announcement to the effect that Dr. Davis not only held forth at the meeting of the American Medical Association at Atlantic City on the evils of kissing, but actually proposed a resolution that kissing should be placed under legal restraint. The possibility of the transmission of disease by means of kissing is a perfectly legitimate subject for discussion, and one to which it is well to draw the attention of the public. It has probably occurred to most medical men, in the course of their practice, to repeatedly warn the fond parent or other dear relative of the danger of infection in this way in cases of tuberculosis, diphtheria, and other infectious diseases. There is no doubt also that the promiscuous osculation which is so customary among women is altogether undesirable, and not without danger to health. But that a resolution was seriously proposed to a meeting of medical men that this habit of kissing should be put under legal restraint is altogether too "Gilbertian" for credence, and we shall therefore not be surprised to learn that this report is also to be placed in the category of sensational fictions of the daily Press.

When these recommendations of the committee were made known some months back, they were somewhat adversely criticised by some of our contemporaries. It was contended that desirable as they might be in theory, they would cause considerable trouble to those medical schools the hospitals of which did not possess lying-in wards, and would involve the medical student in considerable further expense by compelling him to seek the necessary course of instruction in special lying-in institutions. On these grounds it was held that the recommendations were impracticable. On the other hand, we took the view that if the Council was convinced that the present teaching of midwifery was insufficient, their plain and obvious duty was to institute such changes as would ensure efficient practical instruction, and that ways and means must be found of carrying such changes into effect. We consider it, therefore, in every way satisfactory that the Council has adopted the recommendations of the committee, in spite of any difficulties which may be encountered in giving effect to them. The proper and efficient treatment of the mother and child during confinement is one of the most sacred duties of the medical practitioner, and it is a question of the most supreme national importance that he should be thoroughly equipped to carry out that duty before he receives his diploma from a licensing body.

One of the most important matters which came up for discussion during the recent May Session of the General Medical Council was the report of the Practical Midwifery Committee. This committee was formed owing chiefly to the representations of Sir John Williams. He was impressed with the inadequacy in the teaching of midwifery, owing to the fact that whereas the application of modern surgical methods in the lying-in hospitals has resulted in the abolition of puerperal fever within them, no such corresponding change has taken place in midwifery practice outside the hospitals. After careful consideration of the question and consultations with several of the licensing and teaching bodies, the committee made the following recommendations:—

(1) Every student, before commencing the study of practical midwifery, shall be required to have held the offices of clinical medical clerk and surgical dresser, and to have attended a course of lectures on surgery and midwifery. (2) Every student shall be required either (*a*) to have regularly attended the indoor practice of a lying-in hospital or the lying-in wards of a general hospital for a period of three months; and after having received therein practical instruction in the conduct of labour, under the personal supervision of a medical officer, to have conducted 20 cases of labour under official medical supervision; or (*b*) to have conducted not less than 20 cases of labour, subject to the following conditions: That he has, during one month, given regular daily attendance upon the indoor practice of a lying-in hospital or the lying-in wards of a general hospital; and that he has therein conducted cases of labour under the personal supervision of a medical officer of the hospital, who shall, when satisfied of the student's competence, authorise him to conduct outdoor cases under official medical supervision.

In the course of discussion many amendments were moved, but only one, that of Dr. Langley Browne, was adopted, extending the power of instruction to Poor-law infirmaries. Otherwise these recommendations have been adopted by the Council as they stand.

